# Electrochemical Controlling of Double Microgel Layer Formation on an Electrode Surface via an Electrosensitive Inclusion Complex

**DOI:** 10.1021/acsmaterialsau.4c00118

**Published:** 2024-10-29

**Authors:** Kamil Marcisz, Mosayeb Gharakhloo, Damian Jagleniec, Jan Pawlowski, Jan Romanski, Marcin Karbarz

**Affiliations:** † Faculty of Chemistry, University of Warsaw, 1 Ludwika Pasteura Str., PL 02-093 Warsaw, Poland; ‡ Biological and Chemical Research Center, University of Warsaw, 101 Żwirki i Wigury Av., PL 02-089 Warsaw, Poland; § Faculty of Physics, University of Warsaw, 5 Ludwika Pasteura St., PL 02-093 Warsaw, Poland

**Keywords:** environmentally sensitive microgels, electro-reversible host-guest inclusion complex, electroactive gels, monolayer on the electrode surface, double microgel layers

## Abstract

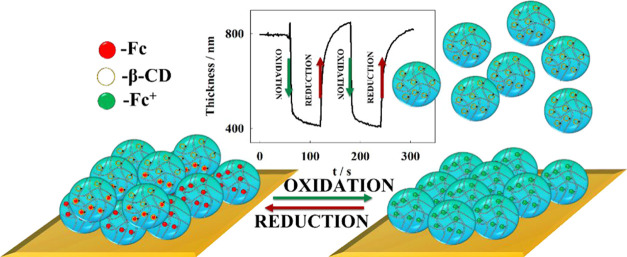

In this study, we demonstrate the formation of a self-assembled microgel double layer on an electrode surface, utilizing the ability to form electro-responsive, reversible inclusion complexes between microgels modified with ferrocene and β-cyclodextrin in these systems. The bottom layer was based on microgels containing ferrocene moieties and derivatives of cysteine. The presence of the amino acid derivative enabled the formation of the well-packed monolayer on the gold surface through chemisorption, while ferrocene was responsible for electroactivity. The addition of βCD-modified microgel led to the formation of the second monolayer, ultimately creating the double layer. Our investigation focuses on the electrochemically controlled formation and deformation processes of the double microgel layer.

## 1 Introduction

Microgels are a class of soft materials consisting of polymer networks filled with a solvent, predominantly water. Some microgels have the ability to undergo a reversible volume phase transition, significantly altering their size in response to alterations in physical or chemical environmental conditions such as temperature, pH, or the presence of specific ions. During the volume phase transition, microgels transition from a swollen to a shrunken state, while concurrently expelling water from the polymer network.^1−3^ Thermoresponsive microgels, particularly those based on poly(*N*-isopropylacrylamide) (pNIPA), belong to this category.^4^ Microgels can be easily modified by incorporating additional functional compounds into the polymer network, such as electroactive probes, thereby imparting redox properties. Their small size and their sensitivity to changes in environmental conditions makes these materials useful in various fields, including drug delivery systems, molecular recognition systems and sensors.^5−15^ Additionally, modifying electrode surfaces with a microgel layer has proven useful in developing electrochemical sensors/biosensors and ON/OFF switch-able electrodes.^16−23^

At the same time, there has been a growing interest in modifying gel polymer networks with cyclic oligosaccharides, such as cyclodextrins.^24−27^ Due to their unique properties, cyclodextrins have also found application in various fields, such as drug delivery systems and specific sensors.^28,29^ Their capacity to form inclusion complexes with hydrophobic species, such as ferrocene, has proven particularly valuable. For example, these host-guest interactions contribute to the acquisition of self-healing properties in hydrogel networks.^30^ The formation of inclusion complexes between cyclodextrin and ferrocene can be reversibly controlled depending on the oxidation state of ferrocene. Oxidation of ferrocene leads to the formation of ferrocene cation, which is much more hydrophilic and results in the dissociation of the complex.^31^ This phenomenon has been utilized in degradable microgel systems for doxorubicin release.^32^ Those electro-reversible host-guest inclusion complex formation was also used to obtain self-assembled layers, based on the modified linear polymers, on the quartz crystal microbalance with dissipation (QCM-D) electrode surface. It was found that electrochemically oxidation of ferrocene groups led to complex deformation with simultaneously top layer desorption.^33−36^

In this study, we present an electrochemical system based on two types of thermoresponsive microgels: one containing ferrocene moieties and the other modified with β-cyclodextrin (βCD). We investigated the influence of electrochemically induced formation/deformation of reversible host-guest inclusion complexes between these two microgels on their interaction and the reversible formation of double microgel layers.

## 2 Experimental Section

### 2.1 Materials

*N*-isopropylacrylamide (NIPA), *N*,*N*′–methylenebisacrylamide (BIS), sodium acrylate (SA), potassium persulfate (KPS), *N*-hydroxysuccinimide (NHS), *N*-(3-dimethylaminopropyl)-*N*′-ethylcarbodiimide hydrochloride (EDC), amineferrocene and β-cyclodextrin (βCD) were obtained from Aldrich. Sodium nitrate (NaNO_3_) was sourced from POCh. All chemicals were used as supplied, except for NIPA, which was recrystallized from a toluene/hexane mixture (30:70 v/v). The *N*,*N*′-bisacryloylcystine (BISS) was synthesized following a method described in the literature.^37^ All solutions were prepared using high-purity water obtained from a Milli-Q Plus/Millipore purification system (water conductivity: 0.056 μS cm^–1^).

### 2.2 Synthesis of Poly(*N*-isopropylacrylamide) Copolymerized with Sodium Acrylate and Cross-Linked with *N*,*N*′-Bisacryloylcystine [p(NIPA-BISS-SA)] Microgels

**Figure 1:**
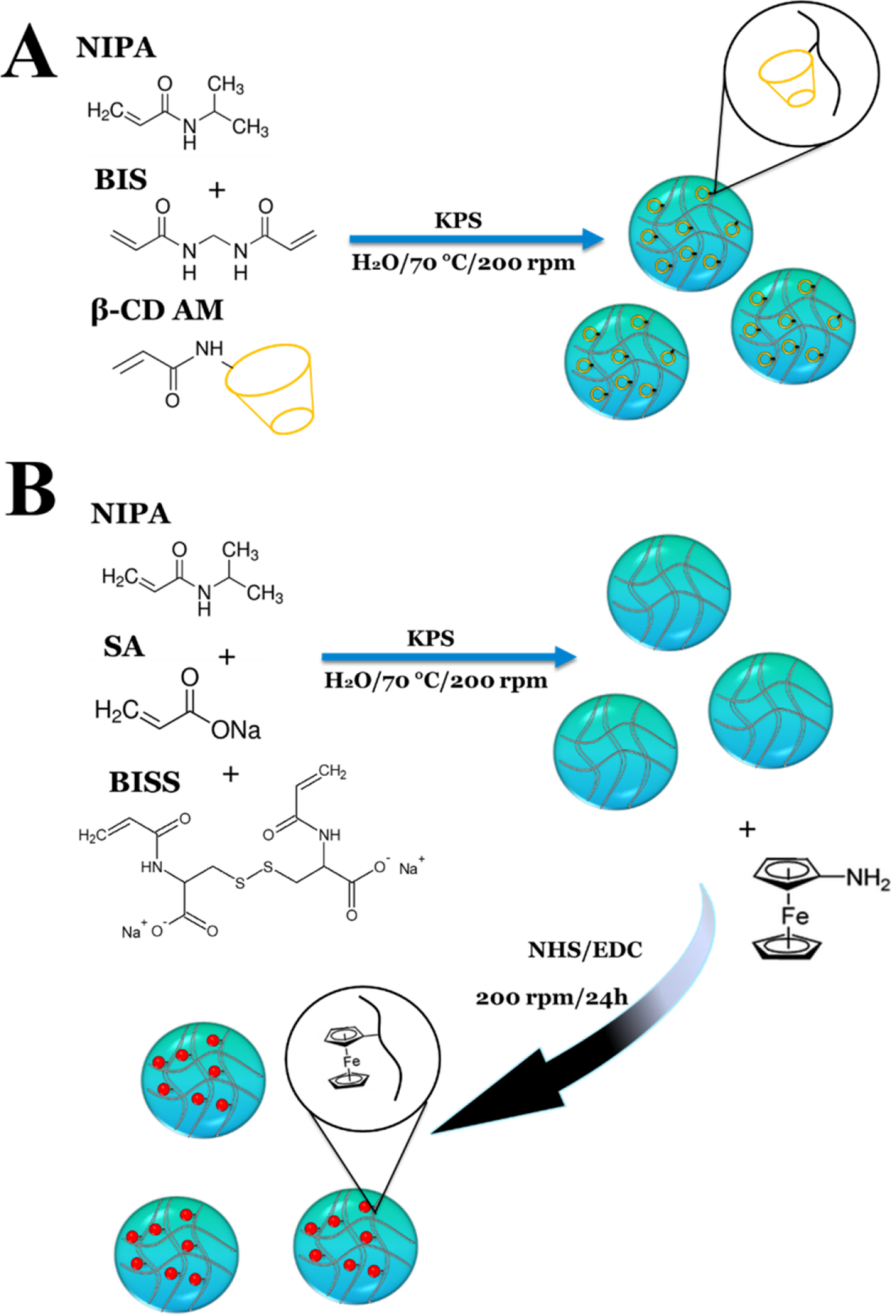
Scheme of the synthesis of p(NIPA-BIS-βCD) (A) and p(NIPA-BISS-Fc) (B) microgels.

The p(NIPA-BISS-SA) microgel was synthesized via a precipitation polymerization reaction.^38^ A mixture of the monomers NIPA, BISS (as a cross-linker) and SA was prepared by dissolving them in 50 mL of deionized water in a two-neck flask, equipped with a magnetic stirrer (set to 200 rpm), a reflux condenser, and inlets for inert gas flow. The overall monomer concentration was adjusted to 70 mM. The microgel was formed with the molar ratios of NIPA, SA and BISS in the pregel solution set to 90, 7, and 3%, respectively. To remove oxygen, the monomer solution was degassed using argon for 30 min before being heated to 70 °C. The polymerization was initiated by adding 27 mg of KPS, dissolved in 5 mL of deionized, degassed water, to the monomer solution. The reaction was conducted under an argon atmosphere at 70 °C for 3 h. Afterward, the solution was allowed to cool to room temperature. Figure 1A provides a schematic illustration of the synthesis process, including the structural formulas of the monomers and the final microgel polymer network. The microgel solutions were purified through dialysis. Each sample was placed in a Spectra/Por dialysis membrane with a molecular weight cutoff of 10 kDa. Dialysis was conducted over 7 days at room temperature in 5 L of water, with daily water changes.

### 2.3 p(NIPA-BISS-SA) Microgel Polymer Network Modification with Aminoferrocene

The p(NIPA-BISS-SA) microgel polymer network was functionalized using aminoferrocene through a reaction facilitated by the coupling agents NHS and EDC. This process resulted in the formation of amide bonds between the amino groups of the aminoferrocene and the carboxyl groups present in the polymer chains of the microgel. The experimental procedure followed was previously reported.^20^ In brief, 5 mL of a p(NIPA-BISS-SA) microgel solution was transferred into a 10 mL flask, after which 1 mL of a solution containing 0.2 mmol of NHS and 1.2 mmol of EDC was added. Subsequently, 0.04 mmol of aminoferrocene, dissolved in 2 mL of a 3:1 water/DMSO mixture, was introduced into the microgel solution. The mixture was stirred at 200 rpm for 24 h at room temperature. To purify the modified microgel, dialysis was performed using 5 L of water over the course of 3 days, with daily water changes.

### 2.4 Synthesis of Acrylamido-β-cyclodextrin Monomer

6-amino-βCD was synthesized following a previously established method.^39^ A 50 mL solution of NaHCO_3_ (11.9 M) was prepared, into which 681 mg (0.6 mmol) of 6-amino-βCD was dissolved. The pH of the solution was adjusted to 10 by adding a concentrated NaOH solution. Subsequently, 97 μL (1.2 mmol) of acryloyl chloride was added dropwise to the mixture while stirring in an ice bath. The reaction was continued for 5 h, after which the mixture was left at room temperature overnight. The resulting reaction mixture was concentrated and poured into 700 mL of acetone, causing the formation of a white precipitate, which was then filtered off. The crude product was purified by silica gel column chromatography using an acetonitrile/water mixture (2:1 v/v), yielding 576 mg (0.48 mmol, 80%) of 6-acrylamido-β-CD as a white solid. The NMR spectrum of the synthesized 6-acrylamido-βCD monomer is shown in Supporting Information (SI) Figure 1.

### 2.5 Synthesis of poly(NIPA Copolymerized with βCD-Am and Cross-Linked with BIS) [p(NIPA-BIS-βCD)] Microgels

The p(NIPA-BIS-βCD) microgel was synthesized using the same procedure as p(NIPA-BISS-SA). Briefly, the monomers NIPA, BIS, and βCD-Am were dissolved in 50 mL of deionized water inside a two-neck flask equipped with a magnetic stirrer (set at 200 rpm), a reflux condenser, and an inert gas inlet and outlet. The total monomer concentration was maintained at 70 mM. The mole fractions of NIPA, βCD-Am, and BIS in the pregel solution were set at 92, 7, and 1%, respectively. The solution was degassed by bubbling argon through it for 30 min to eliminate oxygen, then heated to 70 °C. To initiate polymerization, 27 mg of KPS dissolved in 5 mL of deionized and degassed water was added to the monomer mixture. The reaction proceeded under an argon atmosphere for 3 h at 70 °C. Afterward, the solution was allowed to cool to room temperature. The microgel was purified by dialysis in Spectra/Por dialysis bags (10 kDa molecular weight cutoff) over 7 days at ambient temperature, using 5 L of water, with daily water changes.

### 2.6 Functionalization of the Au QCM-D Electrode Surface with a p(NIPA-BISS-Fc) Monolayer

The p(NIPA-BISS-Fc) microgel was immobilized on the electrode surface via chemisorption, following a previously reported method.^10,40^ In brief, the QCM-D electrochemical cell, which contained an Au QCM-D electrode, was filled with 200 μL of water, with the temperature set to 40 °C. After baseline stabilization, 3 mL of the p(NIPA-BISS-Fc)p(NIPA-BISS-Fc) microgel water solution (concentration: 1.4 mg/mL) at 40 °C was added to the cell and maintained for 12 h. Afterward, the microgel solution was removed, and the electrode coated with p(NIPA-BISS-Fc) was rinsed with water to eliminate any unbound microgel particles. As a result, the Au QCM-D electrode surface was successfully coated with a densely packed microgel monolayer.

### 2.7 Instrumental

#### 2.7.1 Dynamic Light Scattering

(DLS) was employed to determine the hydrodynamic diameter of microgel particles in diluted aqueous dispersions, using a Malvern Zetasizer Nano ZS (U.K.). A 4 mW He-Ne laser with a wavelength of 632.8 nm served as the light source, and measurements were taken at a scattering angle of 173°. Prior to measurements, the solutions were equilibrated at the desired temperatures for 5 min.

#### 2.7.2 Scanning Electron Microscopy (SEM)

images were captured using a Zeiss Merlin field-emission microscope. Initially, the microgel samples were thoroughly dried in a hot-air oven at 50 °C. Subsequently, a thin coating of approximately 3 nm of Au-Pd alloy was applied to the samples using a Polaron SC7620 mini sputter coater.

#### 2.7.3 Scanning/Transmission Electron Microscopy (S/TEM)

For S/TEM, gel samples were prepared by depositing a drop of the aqueous suspension containing microgel and microcomposite particles onto a Formvar-coated copper grid, followed by air drying. The samples were examined using a TALOS F200X microscope (Thermo Scientific), which is equipped with advanced energy dispersive X-ray spectroscopy (EDS) for signal detection and allows for 3D chemical characterization through compositional mapping.

#### 2.7.4 AFM Investigation

Atomic force microscopy (AFM) measurements were carried out using a Dimension Icon microscope equipped with a NanoScope 6 controller (Bruker Corporation, Billerica, MA). Topographical images were acquired in Peak Force Tapping mode, utilizing Bruker RTESPA-150 probes made from antimony n-doped silicon (with a nominal spring constant of 5 N/m and a resonance frequency of 150 kHz). Prior to each experiment, the exact spring constant was determined using the Sader method. Additionally, the deflection sensitivity of the cantilevers and the curvature radius of the probes were assessed using specific calibration samples and procedures. All measurements were performed in air at a stable temperature of 22 ± 1 °C.

#### 2.7.5 Fourier Transform Infrared (FT-IR) Measurements

The FT-IR spectra were recorded with Nicolet iN10-MX-FTIR (Thermo Scientific) spectrophotometer. The microgel sample was measured with the smart iTR attenuated total reflection (ATR) accessory with the diamond crystal. Small microgel solutions portion were dropped on the crystal and heated to evaporate the solvent.

#### 2.7.6 NMR Measurements

Proton (^1^H) NMR spectra used for product characterization were obtained using a Bruker 300 spectrometer. Before analyzing the microgel samples, the purified microgel solution was lyophilized and then dissolved in D_2_O for NMR analysis.

#### 2.7.7 Electrochemical Measurements

Electrochemical measurements were conducted using a CH-Instruments model CHI 400B potentiostat, with control provided by the manufacturer’s software. A three-electrode setup was employed for the experiments. The counter electrode was a platinum wire, while a saturated silver chloride (Ag/AgCl/sat. KCl) electrode served as the reference electrode. For the working electrodes, both a glassy carbon (GC) rod (3 mm in diameter and 37 mm in length) and an Au QCM-D electrode (11.1 mm in diameter, with a working surface area of 0.97 cm^2^) were utilized. The electrodes were positioned either in a glass cell or in an electrochemical QCM-D cell. To reduce electrical interference, the electrochemical cell was housed within a grounded Faraday cage.

#### 2.7.8 Quartz Crystal Microbalance with Dissipation (QCM-D) Measurements

QCM-D studies were conducted using a QEM 401 system from Q-Sense (Biolin Scientific), featuring 4.95 MHz AT-cut gold-coated quartz crystals and operated with the manufacturer’s software. Prior to the deposition, the QCM-D Au electrode was treated with a hot Piranha solution to eliminate organic contaminants, followed by rinsing with water and drying with ethanol. The treated electrode was then positioned in the electrochemical cell provided by the manufacturer. Data obtained from the QCM-D measurements were analyzed using Dfind software, which employs a viscoelastic Voigt-based model to calculate the layer thickness. The assumed parameters for fitting were used as follows: microgel film density −1020 g/dm^3^, fluid density −998 g/dm^3^ and fluid viscosity −1.01 mPa·s.

## 3 Results and Discussion

Two types of thermoresponsive microgels were obtained using the precipitation polymerization method. One type contained built-in ferrocene moieties, while the other contained cyclodextrin groups. The synthesis scheme for both microgels is illustrated in Figure 1A,B. Detailed procedures for the synthesis of the microgels and functional monomers, as well as their characterization, are presented in the Section 2. In a previously reported study, we described the modification of a microgel based on NIPA and sodium acrylate, cross-linked with a diacrylate derivative of cystine (BISS), with ferrocene moieties.^10^ Briefly, carboxylic groups in the microgel polymer network were modified with amineferrocene through amide bond formation in the presence of coupling reagents.

**Figure 2:**
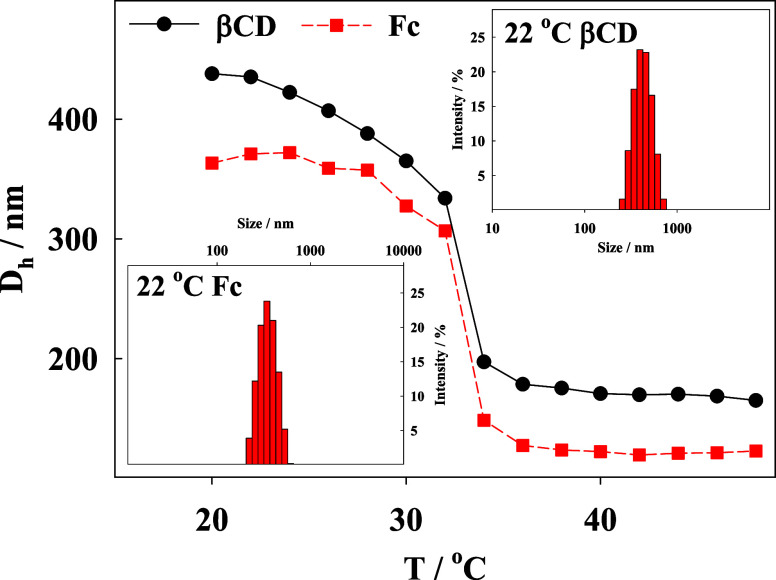
Hydrodynamic diameter changes of p(NIPA-BIS-βCD) (black dots) and p(NIPA-BISS-Fc) (red squares) as a function of temperature. Microgel solution concentrations: p(NIPA-BIS-βCD)–2.6 mg/mL, p(NIPA-BISS-Fc)–1.4 mg/mL. Insets: hydrodynamic diameter distributions of microgel particles modified with ferrocene (Fc) and βCD.

Dynamic light scattering (DLS) was used to examine the temperature-dependent hydrodynamic diameters of the microgel particles. The results, shown in Figure 2 for ferrocene-modified (red squares) and cyclodextrin-modified (black dots) microgel particles, indicate that the swollen state diameters were approximately 440 nm for cyclodextrin-modified microgels and 370 nm for those modified with ferrocene. The microgels are thermoresponsive, with both types exhibiting nearly identical volume phase transition temperatures of approximately 33 °C. Upon transition, the microgels contracted to approximately 170 and 120 nm for β-cyclodextrin-modified and ferrocene-modified microgel, respectively. The morphology of the microgel particles was examined using scanning and transmission electron microscopies, complemented with compositional mapping. The results are presented in SI Figures 2 and 3.

**Figure 3:**
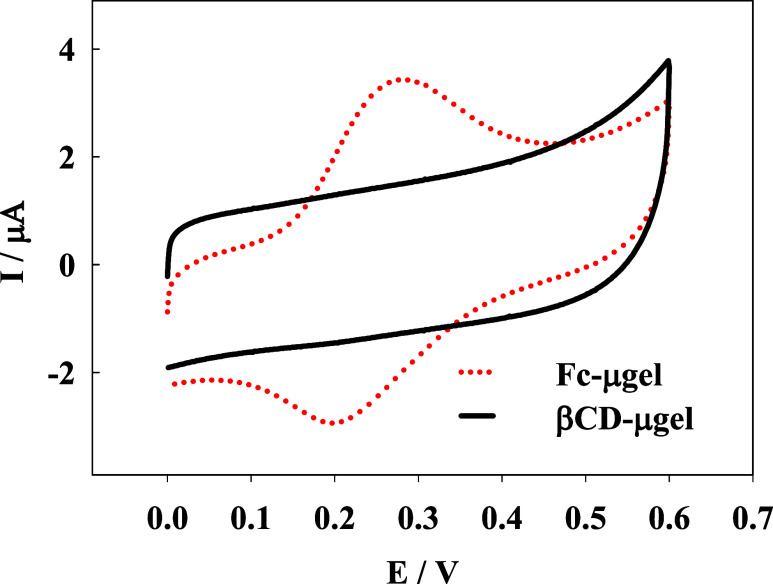
Cyclic voltammograms obtained in p(NIPA-BISS-Fc) (red dotted line) and p(NIPA-BIS-βCD) (black solid line) microgel solutions. Microgel solution concentrations: p(NIPA-BIS-βCD)–2.6 mg/mL, p(NIPA-BISS-Fc)–1.4 mg/mL. Working electrode: GC rod electrode, counter electrode: platinum wire and a saturated silver chloride (Ag/AgCl/sat. KCl) as a reference electrode. Supporting electrolyte: NaNO_3_, 0.01 M, solution volume: 3 mL, scan rates: 50 mV s^–1^.

The electrochemical properties of the microgel solutions were examined using cyclic voltammetry. Figure 3 presents typical voltammograms obtained with a GC rod electrode in solution of p(NIPA-BISS-Fc) (red dotted line) and p(NIPA-BIS-βCD) (black solid line) microgels. The voltammograms obtained from the microgel solution modified with ferrocene groups exhibited characteristic pairs of peaks, while the voltammograms obtained from the βCD-microgel solution showed no electrochemical response (except capacitive currents). These findings confirmed the successful attachment of ferrocene groups to the polymer network. To further confirm the successful incorporation of βCD groups into the microgel polymer network, Fourier-transform infrared (FT-IR) and ^1^H NMR spectroscopies were conducted, revealing characteristic absorption bands for βCD groups and proton signals, as shown in SI Figure 4A,B.^32,41^ Based on the characteristic signals for NIPA and βCD monomers, marked as “a” and “H1” respectively on the ^1^H NMR spectra, the relative amount of βCD groups incorporated into the polymer network of the p(NIPA-BIS-βCD) microgel was estimated to be 20%.

Subsequently, the presence of the S–S bridge in the BISS cross-linker was used to modify the quartz crystal microbalance with dissipation (QCM-D) Au electrode surface with a p(NIPA-BISS-Fc) microgel monolayer through the chemisorption process (see Section 2) as previously described.^10^

**Figure 4:**
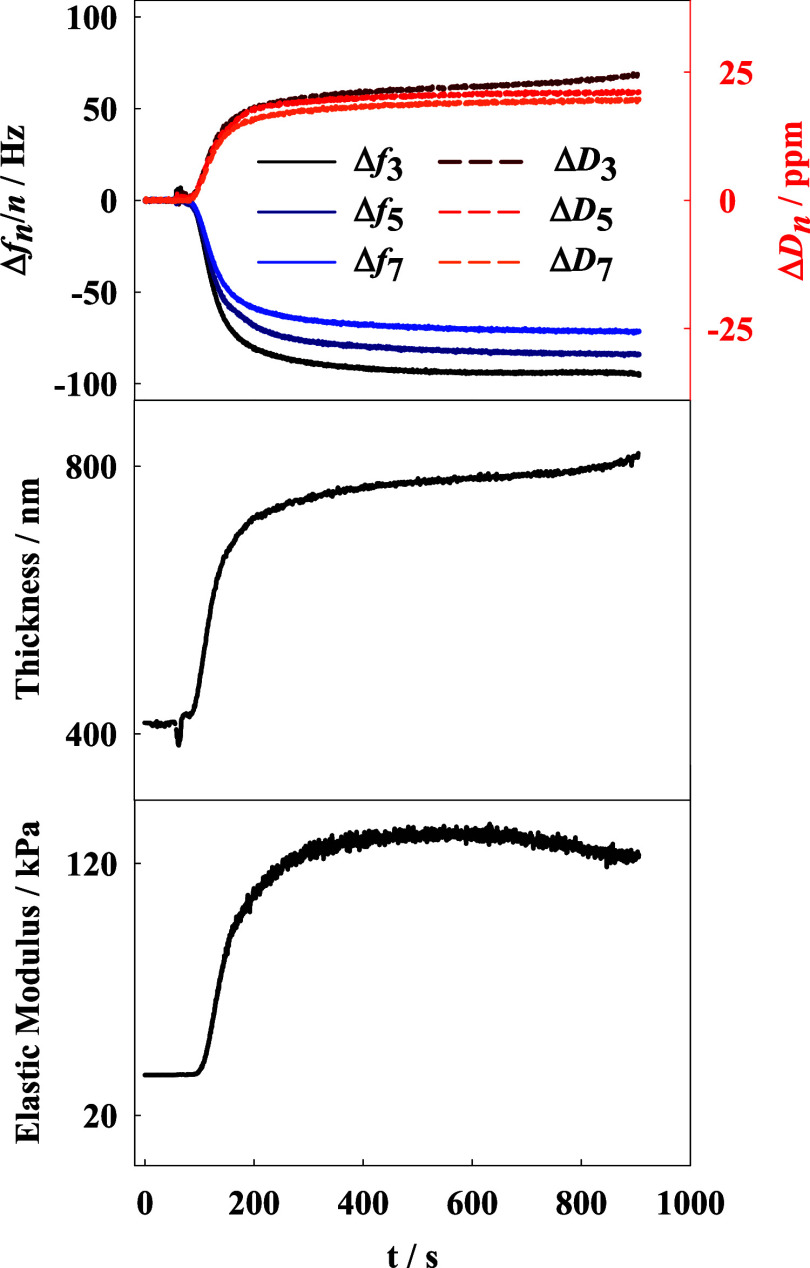
Frequency and dissipation shifts observed upon addition of p(NIPA-BIS-βCD) microgel to p(NIPA-BISS-Fc) microgel monolayer on Au QCM-D electrode surface and calculated: changes in layer thickness and elastic modulus.

Next, the ability to form host-guest complexes between ferrocene groups and βCD groups in the microgel networks was utilized to obtain a double microgel layer on the electrode surface. For this purpose, a portion of p(NIPA-BIS-βCD) microgel water-solution (concentration: 2.6 mg/mL) was added to the QCM-D electrode covered with a p(NIPA-BISS-Fc) microgel monolayer. The frequency and dissipation shifts obtained during the double layer formation are presented in Figure 4. Notably, the addition of the p(NIPA-BIS-βCD) microgel solution caused a decrease in the registered frequency, indicating an increase in mass on the quartz crystal, and an increase in the dissipation shift, indicating an increase in layer softness. The layer thickness, calculated by the manufacturer’s software, is presented in Figure 4. The addition of the p(NIPA-BIS-βCD) microgel was found to cause an increase in layer thickness from approximately 400 nm (for the p(NIPA-BISS-Fc) monolayer) to approximately 790 nm. Some correlation is observed between the obtained monolayer and double-layer thicknesses and the hydrodynamic diameters of the swollen microgels from DLS measurements (Figure 2), suggesting the formation of a well-packed double layer of the microgels.

To rule out the possibility of other nonspecific interactions, additional experiments were conducted: adding a p(NIPA-BIS-βCD) microgel to a bare Au QCM-D electrode and introducing a p(NIPA-BIS) microgel to an Au QCM-D electrode modified with a monolayer of p(NIPA-BISS-Fc) microgel. As can be seen in SI Figure 5, in neither case were significant changes observed in the registered frequency and dissipation shifts.

**Figure 5:**
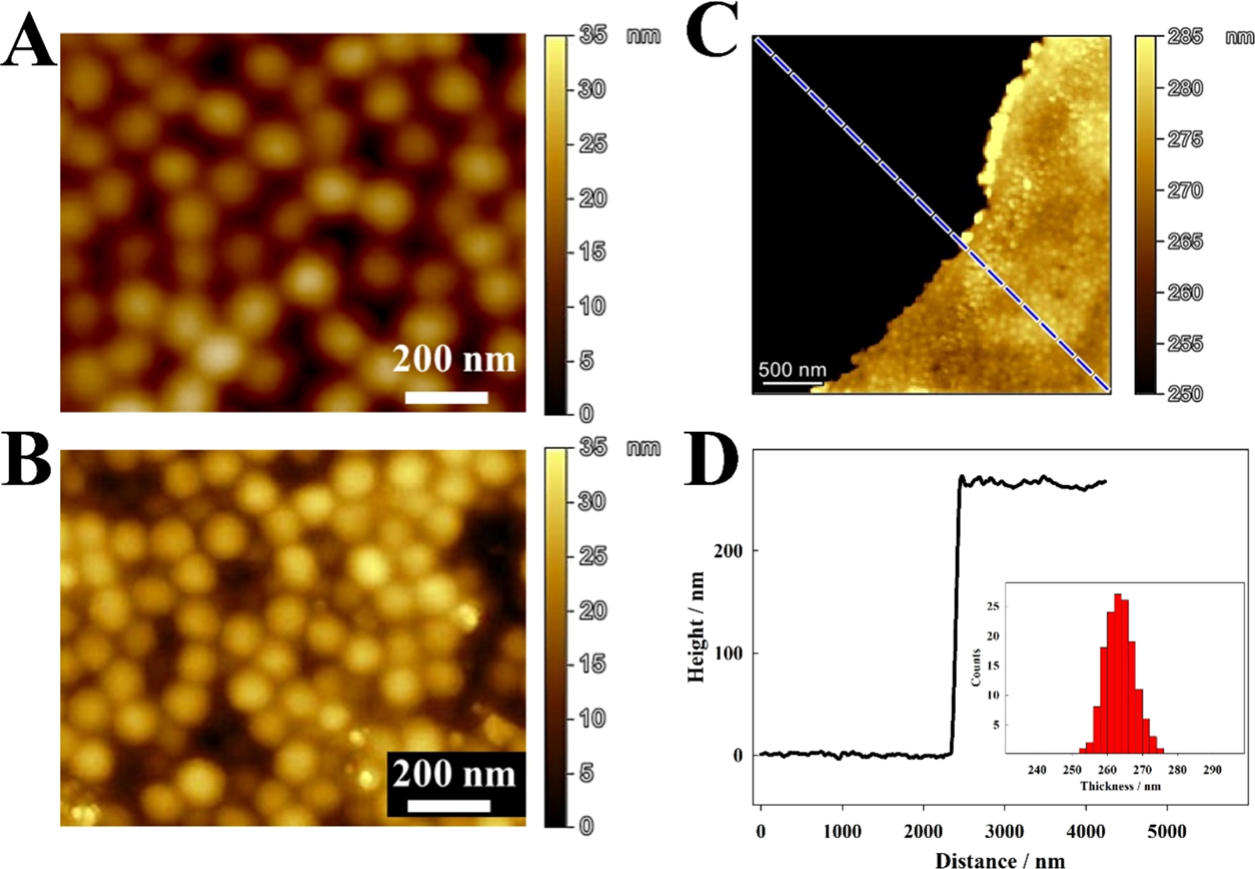
(A) AFM microimage of dry p(NIPA-BISS-Fc) microgel monolayer on the Au QCM-D electrode surface. (B) AFM microimage of the dry double microgel layer on the Au QCM-D electrode surface. (C) Surface topography images for a p(NIPA-BISS-Fc)/p(NIPA-BIS-βCD) microgel double-layer edge. (D) Height-distance profile along the line shown in the AFM image. The inset shows the histogram plot showing the distribution of the p(NIPA-BISS-Fc)/p(NIPA-BIS-βCD) microgel double-layer thickness determined from the height-distance profiles.

The electrodes covered with microgel mono- and double-layer were investigated using the atomic force microscope (AFM). Figure 5A presents an AFM microimage of the dry p(NIPA-BISS-Fc) microgels, showing spherical particles forming a densely packed monolayer on the electrode surface. In contrast, in the microimage obtained for the electrode covered with both types of microgel particles, two layers of spherical particles are observed (Figure 5B).

The surface topography images for the p(NIPA-BISS-Fc)/p(NIPA-BIS-βCD) microgel double-layer edge, along with the obtained height-distance profile (Figure 5C,D), show that the dried double layer thickness was approximately 260 nm, which correlates with the DLS results in their microgel-shrunken states.

**Figure 6:**
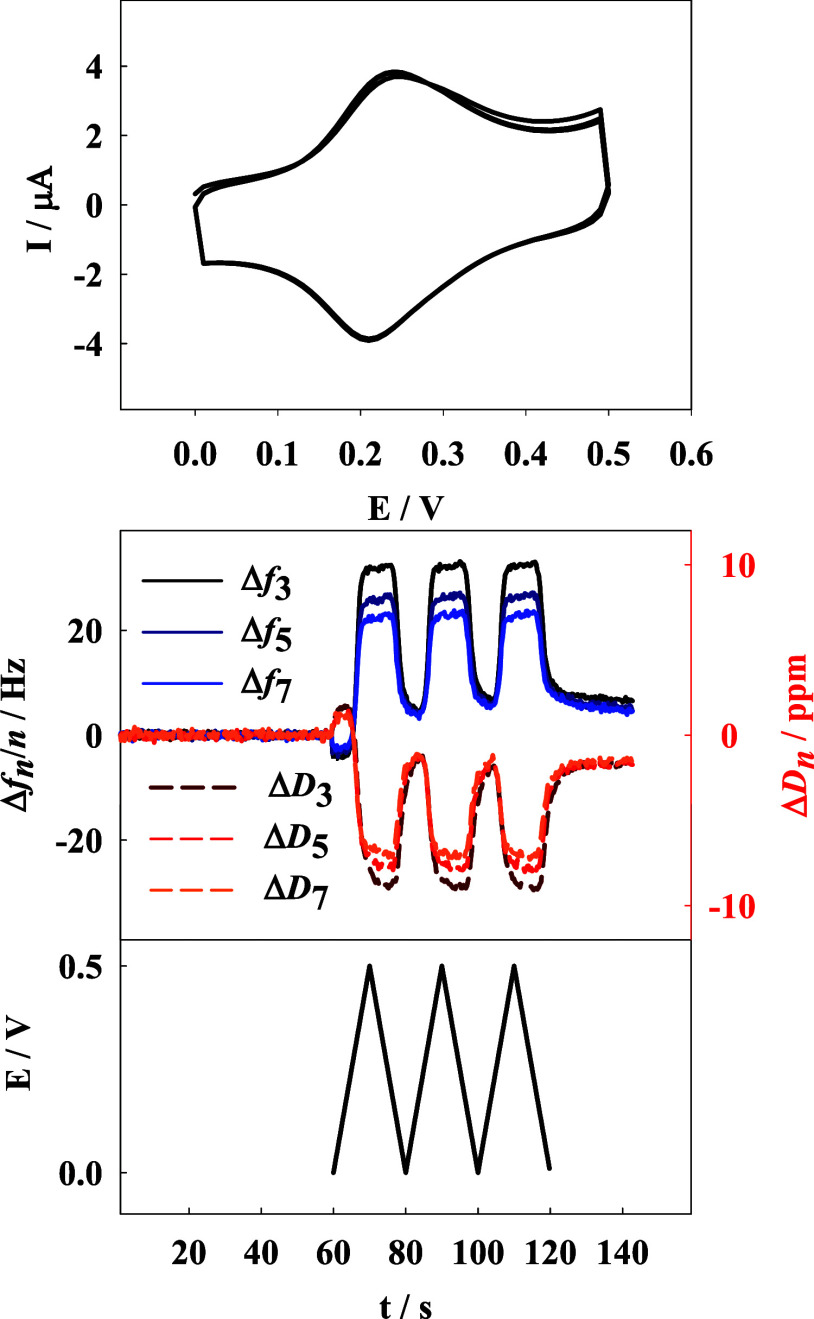
Cyclic voltammograms obtained with Au QCM-D electrode modified with p(NIPA-BIS-βCD)/p(NIPA-BISS-Fc) double layer with simultaneously registered frequency and dissipation shifts. Counter electrode: platinum wire and saturated silver chloride (Ag/AgCl/sat. KCl) as a reference electrode. *T* = 20 °C, supporting electrolyte 0.2 M NaNO_3_, scan rate 50 mV s^–1^.

The formation and interaction of inclusion complexes between ferrocene moieties and βCD moieties is strongly dependent on the oxidation state of ferrocene. In its reduced state, ferrocene is much more hydrophobic than in its oxidized form, allowing for control of these host-guest interactions through the oxidation state of ferrocene. This principle was pivotal in the study of the reversible deformation and formation of double microgel layers on the QCM-D electrode surface, employing a combination of electrochemical techniques and QCM-D. The Au QCM-D electrode covered with a p(NIPA-BISS-Fc)/p(NIPA-BIS-βCD) double layer was examined using cyclic voltammetry. As shown in Figure 6, a pair of peaks characteristic for ferrocene species is visible. The oxidation of ferrocene groups in the p(NIPA-BISS-Fc) microgel led to an increase in the registered frequency shift and a decrease in the dissipation shift, indicating a decrease in mass on the quartz crystal microbalance surface. Conversely, the reduction process of the oxidized ferrocene groups led to the opposite effect: a decrease in frequency shift and an increase in dissipation shift, indicating an increase in mass on the electrode surface.

**Figure 7:**
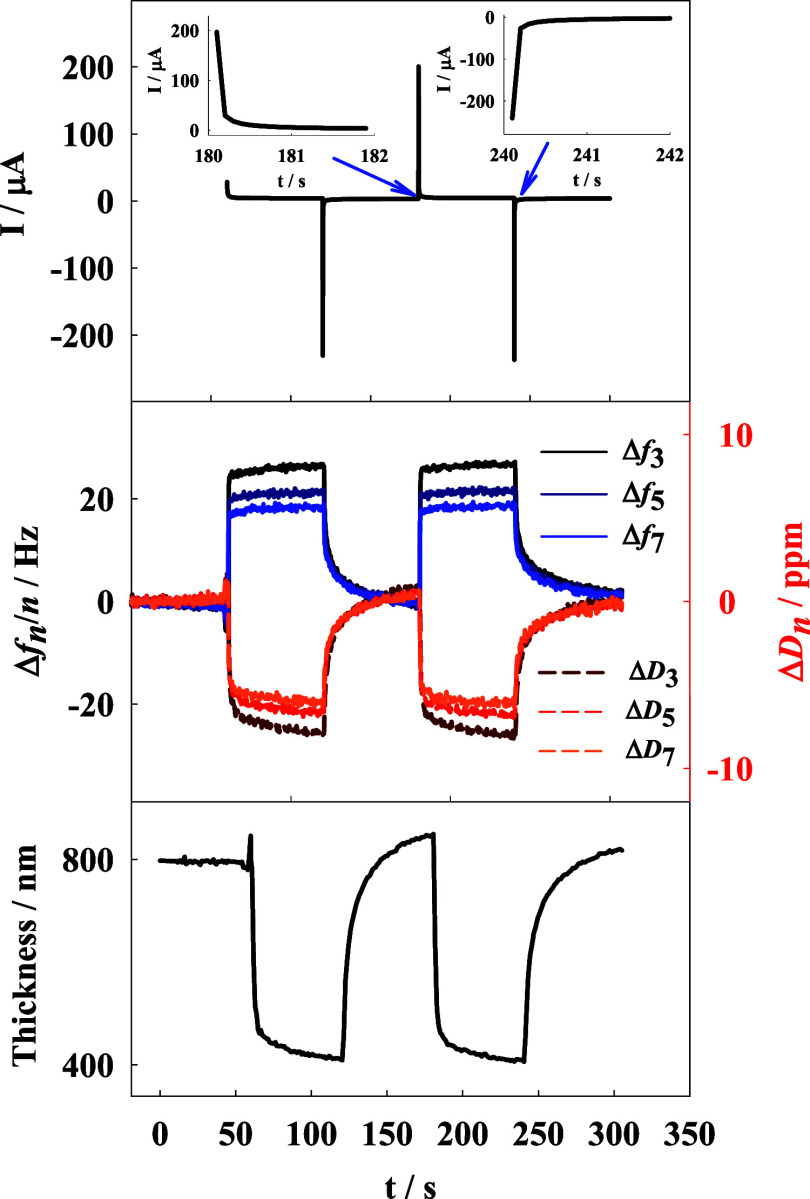
Chronoamperograms obtained with Au QCM-D electrode modified with p(NIPA-BIS-βCD)/p(NIPA-BISS-Fc) double layer with simultaneously registered frequency and dissipation shifts. Counter electrode: platinum wire and saturated silver chloride (Ag/AgCl/sat. KCl) as a reference electrode. *T* = 20 °C, supporting electrolyte 0.2 M NaNO_3_, *E*_ox_ = 0.45 V, *E*_red_ = 0.05 V (60 s potential pulse). Bottom: calculated changes in layer thickness during the electrochemical switching/measurements.

To further investigate the possibility of the detachment of the p(NIPA-BIS-βCD) microgel top layer resulting from the oxidation of ferrocene moieties and the opposite process, chronoamperometry coupled with QCM-D techniques were employed. As illustrated in Figure 7, the application of an appropriate oxidation potential (0.45 V) resulted in an increase in the registered frequency shift, indicative of a decrease in mass on the Au QCM-D electrode surface. Additionally, a decrease in the registered energy dissipation shift was observed, suggesting a changing in the layer’s viscoelastic properties. Calculated from QCM-D data, with a viscoelastic Voigt-based model, the layer thickness changed from approximately 800 nm to approximately 400 nm after applying the oxidation potential to the electrode surface. This change is attributed to the oxidation of ferrocene groups in the p(NIPA-BISS-Fc)/p(NIPA-BIS-βCD) microgel double layer, leading to the dissociation of the inclusion complex and, consequently, the detachment of p(NIPA-BIS-βCD) microgel particles. The calculated layer thickness correlates well with the DLS data (Figure 2), where the sum of microgel hydrodynamic diameters was approximately 810 and 380 nm for p(NIPA-BISS-Fc) microgel particles. This suggests that almost all of the p(NIPA-BIS-βCD) microgel particles were detached from the microgel layer during the oxidation process. Conversely, the reduction of ferrocene cations in the microgel monolayer resulted in the opposite effect: a decrease in frequency and an increase in dissipation shifts, indicating the attachment of the p(NIPA-BIS-βCD) microgel particles to the p(NIPA-BISS-Fc) microgel monolayer on the electrode surface. Both detachment and attachment of the microgel particles to the microgel monolayer on the electrode surface occurred rapidly. These processes can be divided into three steps: the first involves electron transport between the electrode and the first p(NIPA-BISS-Fc) microgel layer, associated with the oxidation/reduction of the ferrocene groups; next, dissociation/formation of inclusion complexes between ferrocene and cyclodextrin moieties occurs; and finally, the detachment/attachment of the p(NIPA-BIS-βCD) microgel particles takes place. The rapid progression of the last process, as measured by the change in the quartz resonator’s frequency, is most likely related to the relatively fast Brownian motion of the gel microparticles. Due to the fact that the QCM electrode in the used setup is placed at the bottom of the cell, the possibility of microgel particle sedimentation should be discussed. In fact, the p(NIPA-BIS-βCD) microgel solution is very stable and does not sediment, even after several months of storage. However, some slippage of certain microgel particles on top of the monolayer is possible. Even if this occurs, it does not disturb the QCM response.

Assuming that the electrolysis process of the gel film during the chronoamperometric measurements was complete, the amount of ferrocene moieties in the microgel layer was calculated based on the charge that flowed during the experiment. After correcting for the capacitive current, which had a significant contribution at the very beginning of applying the potential pulse, the average charge during the oxidation and reduction steps was 5.0 × 10^–5^ C. Based on this value, the content of ferrocene groups in the layer was estimated to be 5.2 × 10^–10^ mol.

In addition, similar QCM-D experiments were conducted for electrodes modified with a monolayer of p(NIPA-BISS-Fc) microgel in a solution without p(NIPA-BIS-βCD) microgel particles (SI Figure 6). However, only slight changes in frequency were observed, suggesting that the oxidation and reduction of the p(NIPA-BISS-Fc) microgel monolayer have no significant influence on the data presented in Figures 6 and 7. These findings are in good agreement with our previous study,^10^ where the influence of the oxidation state of ferrocene groups in the p(NIPA-BISS-Fc) microgel on the degree of swelling was thoroughly analyzed. It was found that the oxidation of ferrocene groups in the p(NIPA-BISS-Fc) microgel did not cause significant changes in the microgel’s hydrodynamic diameter or in the frequency of the QCM electrode modified with the p(NIPA-BISS-Fc) microgel at 20 °C.

## 4 Conclusions

In conclusion, we successfully obtained a double layer of microgels on the Au QCM electrode. The bottom layer was composed of microgels containing ferrocene moieties and derivatives of cysteine. The presence of the amino acid derivative enabled the formation of the well-packed monolayer on the gold surface through chemisorption, while ferrocene conferred electroactivity. The addition of βCD-modified microgel was found to enable the formation of the second monolayer, ultimately creating the double layer. The self-assembly process of forming the second layer was associated with the formation of a host-guest inclusion complexes between ferrocene and βCD groups. The complex formation’s reliance on the oxidation state of ferrocene allowed us to electrochemically control the double layer’s formation. This process of forming and deforming the double layer was found to be both rapid and reversible. This unique capability also allows for the electrochemical control of the release of microgel from a conducting surface into a solution and adsorbing microgel from solutions, thus paving the way for potential applications in advanced drug release systems, sensors, and electrochemically controlled sorption/filtration systems.

## Supporting Information Available

NMR and FT-IR spectra of monomer and microgel, SEM and TEM images of microgels, additional electrochemical and QCM-D results (PDF)
