# Supplementary material for: Electrochemical Controlling of Double Microgel Layer Formation on an Electrode Surface via an Electrosensitive Inclusion Complex
Source: ACS Mater Au. 2024 Oct 29;5(1):191–9. doi: 10.1021/acsmaterialsau.4c00118 (PMC11718541; doi:10.1021/acsmaterialsau.4c00118)
Supplement: Supplementary file 1 — mg4c00118_si_001.pdf [file mg4c00118_si_001.pdf]

Supplementary Information

Electrochemical controlling of double microgel layer formation on an electrode surface via an electrosensitive inclusion complex

Kamil Marcisz[a], Mosayeb Gharakhloo[a], Damian Jagleniec[a], Jan Pawlowski[c], Jan Romanski[a], Marcin Karbarz[a,c,*]

[a] Faculty of Chemistry, University of Warsaw, Pasteura 1, PL 02-093 Warsaw, Poland.

[b] Faculty of Chemistry, Biological and Chemical Research Center, University of Warsaw, 101 Żwirki i Wigury Av., PL 02-089 Warsaw, Poland

[c] Faculty of Physics, 5 Ludwika Pasteura St., PL 02-093 Warsaw, Poland.

* E-mail: karbarz@chem.uw.edu.pl

Results

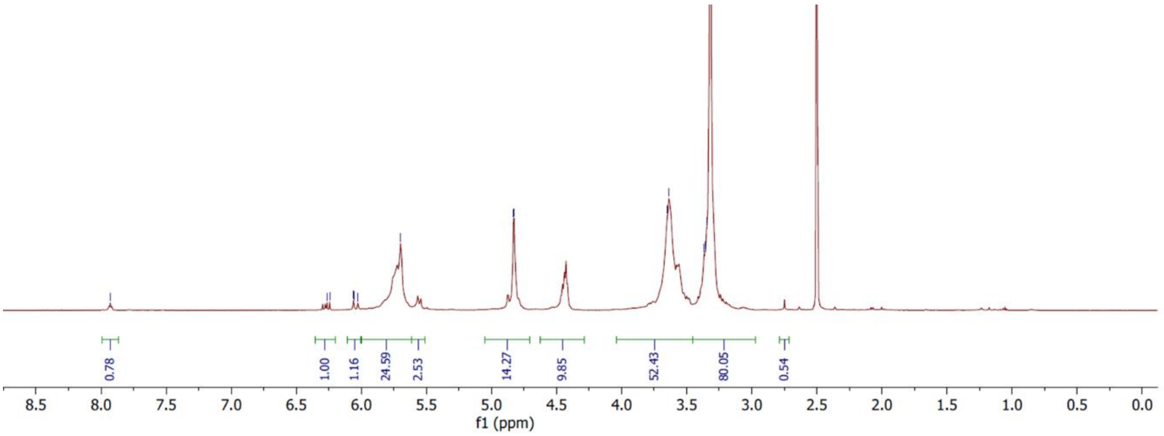

SI Fig. 1. ^{1}H NMR spectra of obtained βCD-Am monomer in DMSO-d_6.

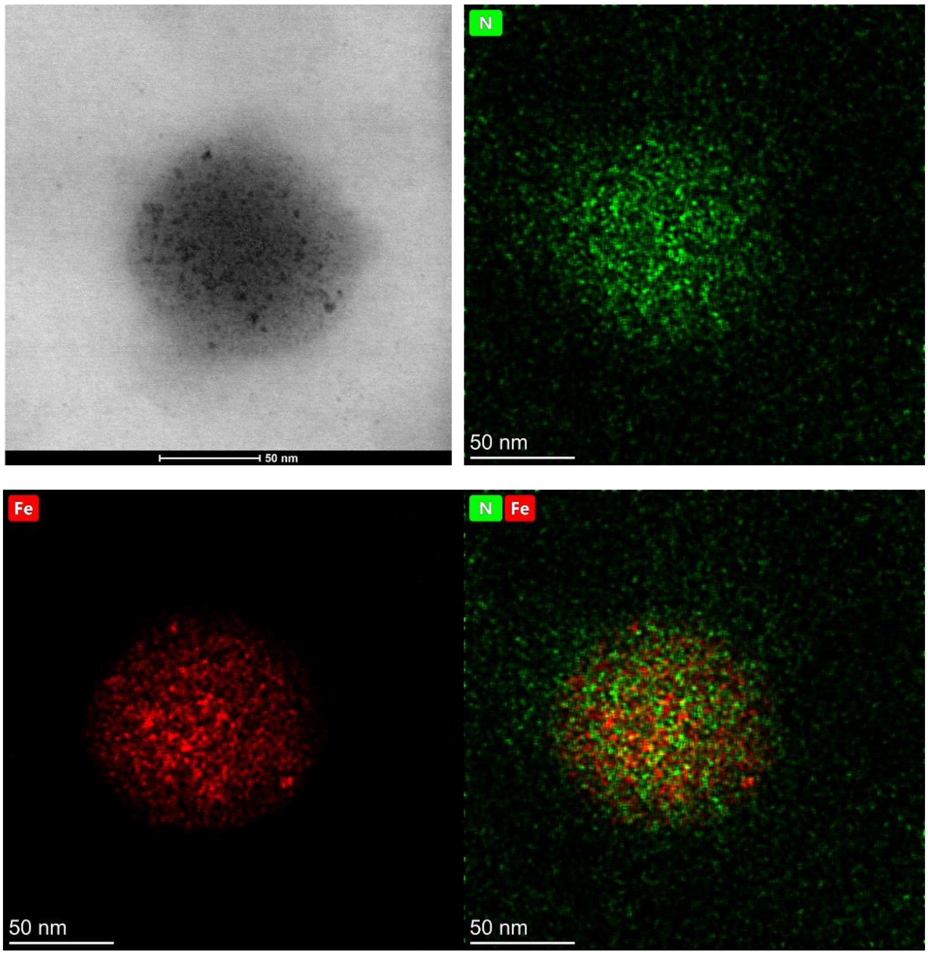

SI Fig. 2. TEM microimage of single p(NIPA-BISS-Fc) microgel sphere and compositional mapping of atoms in that sphere.

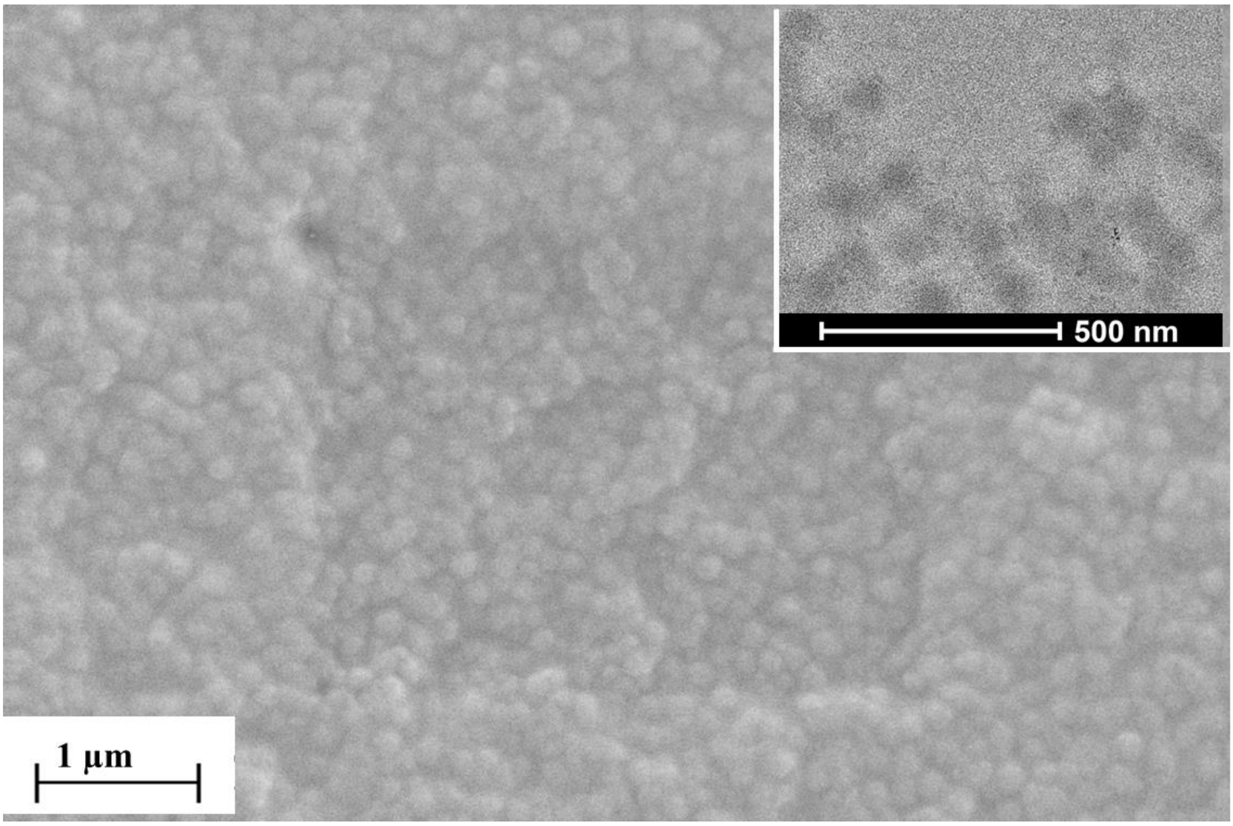

SI Fig. 3. SEM and TEM (inset) microimages of p(NIPA-BIS-βCD) microgel particles.

A

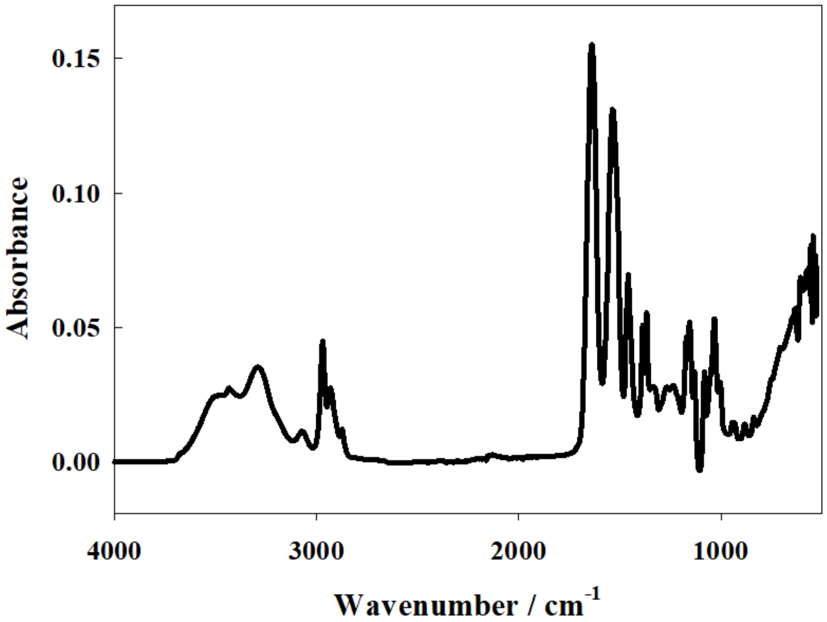

B

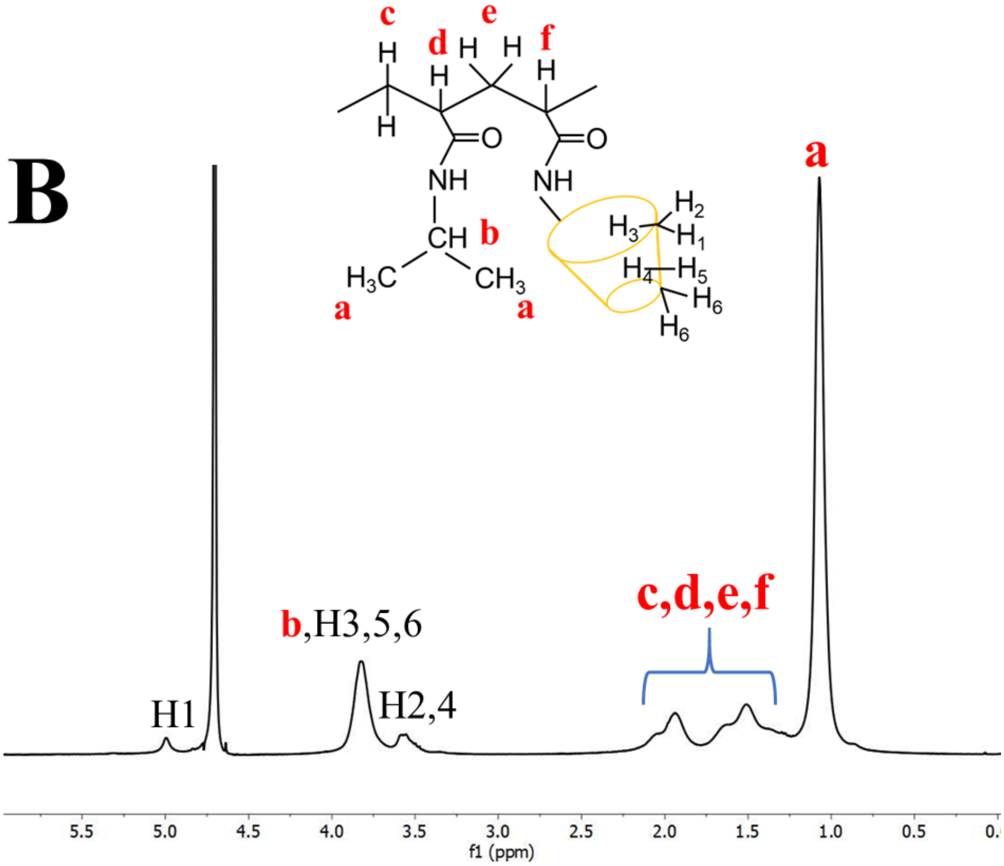

SI Fig. 4. A) FT-IR spectra of p(NIPA-BIS-βCD) microgel sample. Background was obtained with p(NIPA-BIS) microgel sample. B) ^{1}H NMR spectra of p(NIPA-BIS-βCD) microgel sample in D_2O.

A

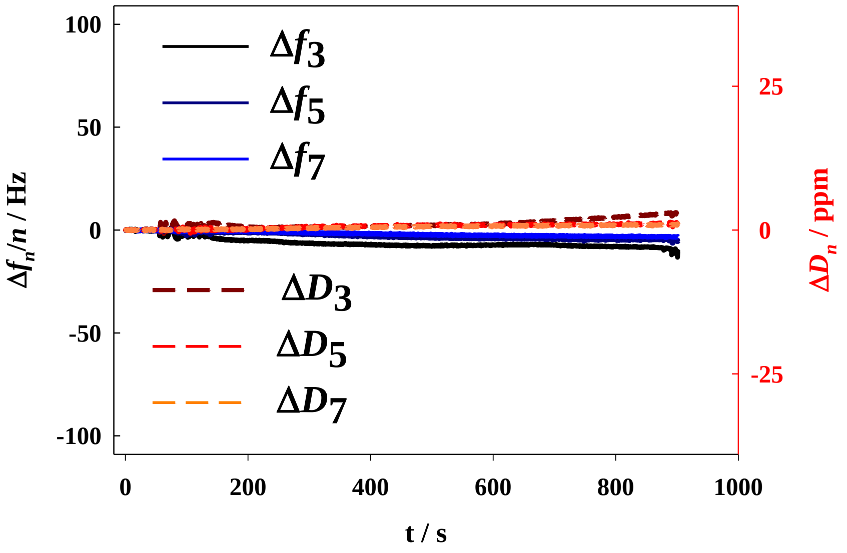

B

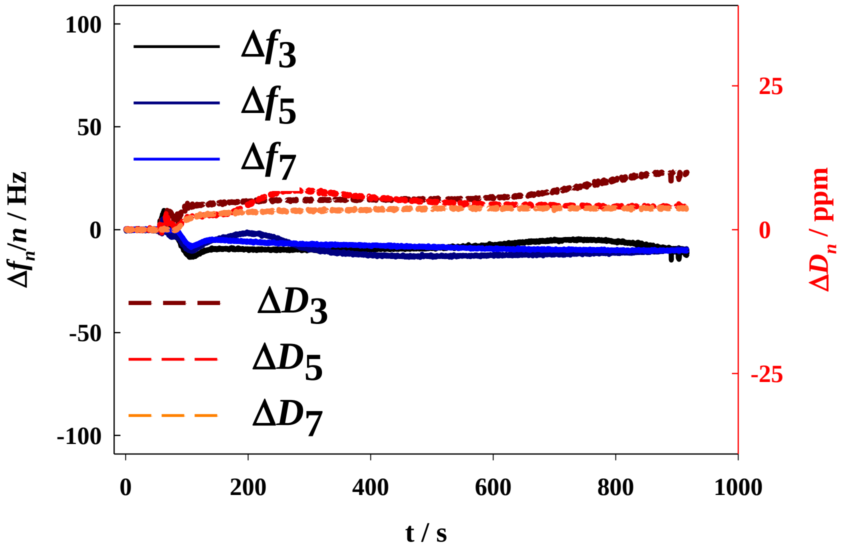

SI Fig. 5. A) Frequency and dissipation shift during p(NIPA-BIS-βCD) microgel addition to the bare Au QCM-D electrode surface. B) Frequency and dissipation shift during p(NIPA-BIS) microgel addition to p(NIPA-BISS-Fc) microgel monolayer on the Au QCM-D electrode surface.

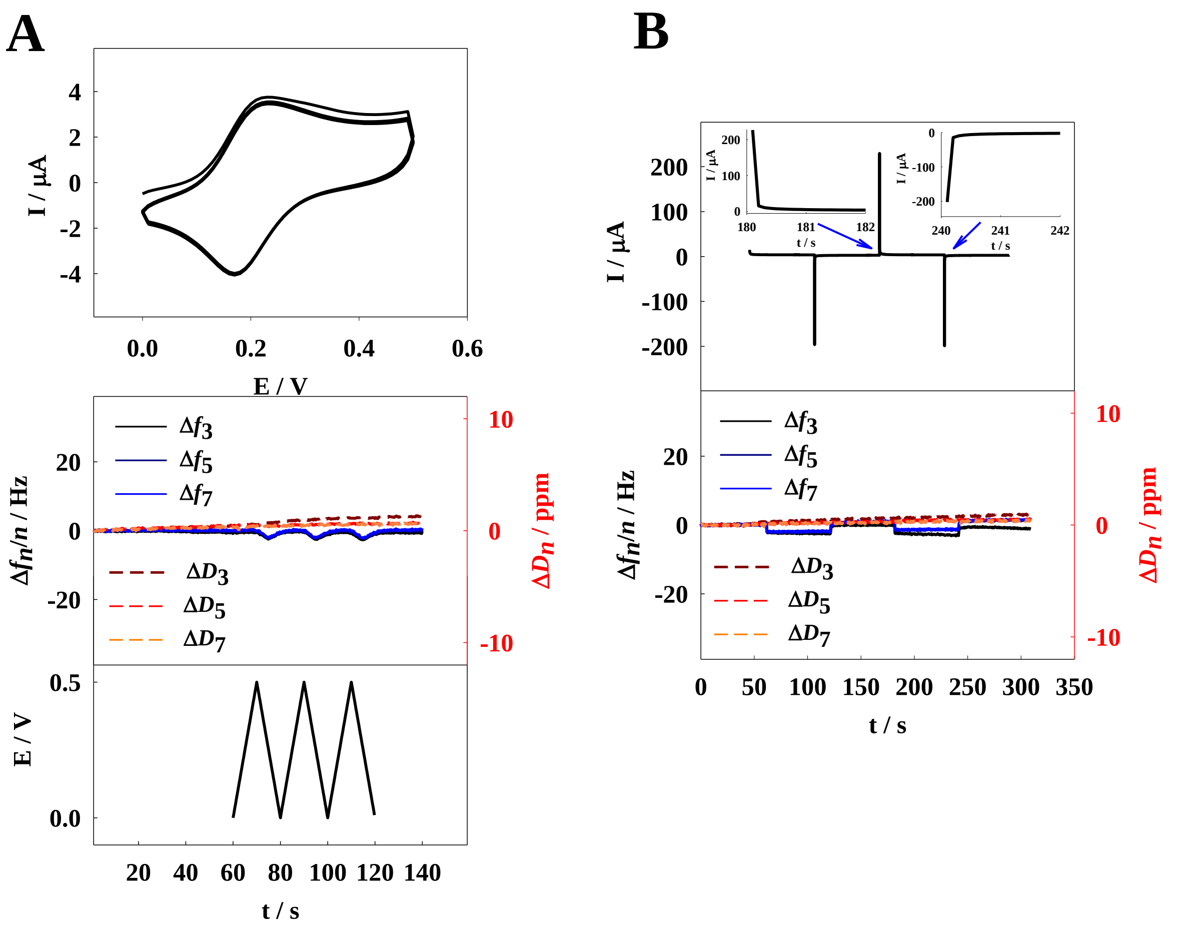

SI Fig. 6. A) Cyclic voltammograms obtained with Au QCM-D electrode modified with p(NIPA-BISS-Fc) monolayer with simultaneously registered frequency and dissipation shifts. T=20 °C, supporting electrolyte 0.2M $NaNO_3$, scan rate 50 $mV \cdot s^{-1}$. B) Chronoamperograms obtained with Au QCM-D electrode modified with p(NIPA-BISS-Fc) monolayer with simultaneously registered frequency and dissipation shifts. T=20 °C, supporting electrolyte 0.2M $NaNO_3$, E_{ox}=0.45 V, E_{red}=0.05 V.
